# Placenta-Targeted Nanoparticles Loaded with PFKFB3 Overexpression Plasmids Enhance Angiogenesis and Placental Function

**DOI:** 10.3390/bioengineering9110652

**Published:** 2022-11-04

**Authors:** Qi Li, Xiaoxia Liu, Weifang Liu, Yang Zhang, Wen Liu, Mengying Wu, Zhirui Chen, Yin Zhao, Li Zou

**Affiliations:** 1 Department of Reproductive Medicine, Xiangya Hospital, Central South University, Changsha 410008, China; 2 Department of Obstetrics and Gynecology, Union Hospital, Tongji Medical College, Huazhong University of Science and Technology, Wuhan 430022, China; 3 Department of Pain Treatment, Union Hospital, Tongji Medical College, Huazhong University of Science and Technology, Wuhan 430022, China

**Keywords:** PFKFB3, nanoparticles, placenta, angiogenesis, pregnancy

## Abstract

Placental angiogenesis disorder and placental dysplasia are important causes of many pregnancy complications. Due to safety and economic benefits, effective treatment strategies are currently limited. PFKFB3 is a key regulator of glycolysis that controls angiogenesis through a metabolic pathway independent of genetic signals. In this study, we constructed the nanodrug T-NP_PFKFB3_ and explored its feasibility to promote angiogenesis and enhance placental function. First, liposomes containing PFKFB3 overexpression plasmids modified by the placental homing peptide CGKRK were synthesized by the thin film method. In vivo experiments revealed that T-NP_PFKFB3_ injected intravenously specifically accumulated in the mouse placenta and therein upregulated the expression of PFKFB3 without affecting its expression in other important organs. In addition, T-NP_PFKFB3_ promoted placental angiogenesis and increased the fetal and placental weights of the mice. Finally, we evaluated the safety of T-NP_PFKFB3_. The expression levels of ALS/AST/BUN in the sera of pregnant mice were not significantly different from those in the sera of control group mice. However, T-NP_PFKFB3_ did not cause obvious fetal abnormalities or alter the average litter size. In conclusion, T-NP_PFKFB3_ can specifically target the placenta, promote angiogenesis, and enhance placental function without obvious side effects. Therefore, it has potential as a new strategy for the treatment of pregnancy complications.

## 1. Introduction

More than 10% of pregnant women develop serious complications during pregnancy, such as preeclampsia, fetal growth restriction (FGR), and placenta implantation, which lead to substantial maternal and infant morbidity and mortality [1]. The underlying causes are complex, but much evidence shows that placental dysfunction plays an important role in these complications, leading to iatrogenic premature delivery [2]. Studies on animal models of pregnancy complications have also shown that enhancing placental function can alleviate maternal symptoms and promote fetal growth [3,4]. However, pregnant women are considered to be a high-risk, low-return group. As a result, only a few drugs have been licensed for the treatment of pregnancy disorders in the past 20 years, and the efficacies of existing drugs are extremely limited [5,6].

Placental angiogenesis is considered to be one of the more important factors influencing pregnancy complications [7]. The proper formation of placental blood vessels ensures nutrient and gas exchange and effectively eliminates fetal waste, while the risk of adverse outcomes in pregnancies with FGR is significantly increased [8]. Angiogenesis is regulated by a series of complex mechanisms. Endothelial cells (ECs) have long been thought to mainly regulate angiogenesis through the vascular endothelial growth factor signaling pathway, but the effect of metabolism has attracted increasing attention in recent years [9]. Recent studies have shown that ECs can also drive angiogenesis through metabolic pathways in parallel with genetic signals [10,11]. In view of the limited success rate of growth factor-targeted regimens, we think that targeting EC metabolism as an alternative method may improve the current problems of limited efficacy and drug resistance.

Although oxygen is immediately available in the bloodstream, ECs rely on glycolysis to provide energy to maintain their normal functions [12], and PFKFB3 is a key regulator of glycolysis in ECs. It produces fructose-2,6-diphosphate (F-2,6-BP), which is isomerized to activate the rate-limiting enzyme PFK-1 [13]. PFKFB3 has been shown to promote vessel sprouting and to play important roles in various organs, such as the heart, liver, and colon [10,14]. In addition, we previously revealed that PFKFB3 is expressed in the placental tissues of pregnant women and that MALAT1 regulates PFKFB3-driven glycolysis by sponging miR-26a/26b, thereby affecting angiogenesis in ECs [15]. Therefore, we speculate that PFKFB3 is a potential target to promote angiogenesis and improve placental function. However, the role of PFKFB3 in the placenta has not been fully elucidated, and further in vivo experiments are needed.

In recent years, nanomaterials have been increasingly used to develop new therapeutic methods, and great progress has been made in the molecular diagnosis and targeted therapies of cancer [16,17]. The main goal of nanomedicine research is to develop strategies for transporting drugs, nucleic acids, biological agents, and other effective loads to targets in vivo by using nanoparticles (NPs) as carriers [18]. At the same time, targeted ligands such as peptides can be bound to the NP surface to promote particle aggregation in specific organs or tissues and reduce their transport to unexpected positions [19]. Because targeted drug delivery has the potential to improve drug efficacy and reduce the risk of systemic administration and nontargeted side effects, nanodrug-based therapies are considered to be useful in the treatment of placental-related diseases, thereby improving maternal and fetal outcomes [20]. In addition, King et al. [21] performed T7 phage screening on pregnant mice and identified the tumor homing peptide CGKRK (Cys-Gly-Lys-Arg-Lys), which was shown to selectively bind to the spiral arteries of the mouse placenta and uterus and did not affect normal development when intravenously injected. In another study, Beards et al. [22] modified NPs with the placenta-specific homing peptide CGKRK and confirmed that a miR-145 inhibitor was specifically delivered to the placenta, thereby increasing the placental and fetal weights in pregnant mice. Therefore, based on the unique advantages of the targeting ability and safety of nanodrugs, we used a liposome vector with the surface-conjugated placenta-targeting peptide CGKRK instead of the commonly used viral vector to deliver PFKFB3 overexpression plasmids to the placenta and to assess whether the complexes could promote placental growth and optimize pregnancy outcomes.

In this study, we designed and synthesized liposomes containing PFKFB3 overexpression plasmids modified by the placental homing peptide CGKRK and verified the feasibility of T-NP_PFKFB3_ to promote angiogenesis and enhance placental function. The results showed that the T-NP_PFKFB3_-targeted enhancement of PFKFB3 expression in the placenta promoted angiogenesis and increased the weights of the mouse fetus and placenta without obvious side effects.

## 2. Materials and Methods

### 2.1. Animal Procedures

C57BL/6J mice were purchased from the Hubei Experimental Animal Research Center (Wuhan, China). The mice were housed in a pathogen-free animal facility with a 12 h light–dark cycle at 21–23 °C and had free access to food and water. The gestational ages of the mice were determined by the presence of a copulation plug (E0.5 = copulation plug day). All procedures in this study were approved by the Institutional Animal Care and Use Committee of Tongji Medical College in Wuhan, China (permit number: 2487).

### 2.2. Synthesis of Nanoparticles

Lipid-polymer NPs loaded with PFKFB3 overexpression plasmids (NP_PFKFB3_) were synthesized from DSPE-PEG2000-MAL, lecithin, and PFKFB3 plasmids using the previously published thin film method [21]. To prepare placenta-targeted liposomes, the synthetic placental homing peptide CGKRK was conjugated to NP (T-NP_PFKFB3_). The PFKFB3 overexpression plasmids were purchased from GenePharma (Shanghai, China), and other materials were purchased from Ruixi Biological Technology (Xi’an, China).

### 2.3. Characterization of Nanoparticles

The size distributions and zeta potentials of the NPs were measured by dynamic light scattering (DLS) using a Zetasizer Nano ZS instrument (Model ZEN3600, Malvern, UK). The stability of NPs in PBS containing10% fetal bovine serum (FBS) was evaluated by examining changes in size.

### 2.4. Nanoparticle Treatment Study

Pregnant C57BL/6J mice were divided into three groups (*n* = 8 per group) and intravenously injected with 100 μL of the control solution (PBS), plain (undecorated) liposomes loaded with PFKFB3 overexpression plasmids, or CGKRK-decorated liposomes via the tail vein on three separate days (E12.5, E14.5 and E16.5). The concentration of the PFKFB3 overexpression plasmid was approximately 0.25 mg/mL. All of the pregnant mice were sacrificed at full term (E18.5), and their tissues were collected for the following assessments: weight and size of the fetus or placenta and size of the litter. Tissues were stored at −80 °C or fixed in 4% formaldehyde for paraffin embedding.

### 2.5. Immunohistochemistry

Immunohistochemistry (IHC) was employed to evaluate the PFKFB3 expression in placental tissues as follows: after rehydration, paraffin sections were heated in citrate buffer (pH = 6.0) for antigen retrieval and blocked with 10% normal goat serum. Thereafter, endogenous peroxidase activity was quenched by incubating the sections with 3% H_2_O_2_, and the sections were then incubated with a PFKFB3-specific polyclonal antibody (1:500; 13763-1-AP; Proteintech), a CD34 antibody (1:200; AF5149; Affinity), or control IgG at 4 °C overnight. Finally, the slides were incubated with HRP-conjugated goat anti-rabbit IgG (1:1000; Santa Cruz, CA, USA) and observed under a light microscope.

### 2.6. RT-qPCR

Total RNA was extracted from mouse tissues using TRIzol Reagent (Vazyme Biotech, Nanjing, China), and cDNA templates were synthesized with a PrimeScript RT reagent Kit (Takara, Tokyo, Japan). RT-qPCR was performed using SYBR Green qPCR Mastermix (DBI, Shanghai, China) with the StepOnePlus Real-Time PCR system (Applied Biosystems, CA, USA). β-actin was employed as the internal control. The PCR primer sequences are shown below:

Mouse PFKFB3-F (5′–3′): CCACCAAAAAGCCTCGCATC 

Mouse PFKFB3-R (5′–3′): ACAGACAGTTCGTAGACAGGC

Mouse β-actin-F (5′–3′): CCGCGAGTACAACCTTCTTG

Mouse β-actin-R (5′–3′): TGACCCATACCCACCATCAC.

### 2.7. Western Blot Analysis

The concentrations of the total proteins extracted from mouse tissues were determined using a BCA protein assay kit (P0010S; Beyotime, Jiangsu, China). The proteins (30 mg) were then subjected to 10% sodium dodecyl sulfate–polyacrylamide gel electrophoresis (SDS–PAGE) and transferred onto polyvinylidene fluoride (PVDF) membranes. Then, the blots were incubated with 5% nonfat milk in Tris-buffered saline containing 0.05% Tween-20 for 1 h at room temperature, and then with a rabbit polyclonal anti-PFKFB3 antibody (1:1000; 13763-1-AP; Proteintech, Chicago, IL, USA) overnight at 4 °C. β-Actin (1:2000; 20536-1-AP; Proteintech) was used as an internal control. After washing with TBST, the membranes were incubated with the appropriate secondary antibodies for 1 h at room temperature, and the proteins were visualized using an ECL kit (WBKLS0500; Millipore, MA, USA).

### 2.8. Measurements of ALT, AST, and BUN Levels

The serum levels of alanine aminotransferase (ALT), aspartate aminotransferase (AST), and blood urea nitrogen (BUN) in mice were measured using the following assay kits according to the manufacturers’ instructions: Alanine Aminotransferase Assay Kit (C009-2-1, Jiancheng, Nanjing, China), Aspartate Aminotransferase Assay Kit (C010-2-1, Jiancheng), and Urea Assay Kit (C013-2-1, Jiancheng).

### 2.9. Statistical Analysis

Statistical analyses were performed using GraphPad Prism 8.0 software (GraphPad Software). Data are presented as the means ± SDs. Student’s *t*-test and one-way ANOVA followed by Dunnett’s multiple comparisons test were used to calculate the differences. *p* < 0.05 was considered significant. All data were obtained from ≥3 independent experiments.

## 3. Results

### 3.1. Synthesis and Characterization of Placenta-Targeted Nanoparticles Loaded with PFKFB3 Plasmids

**Figure 1:**
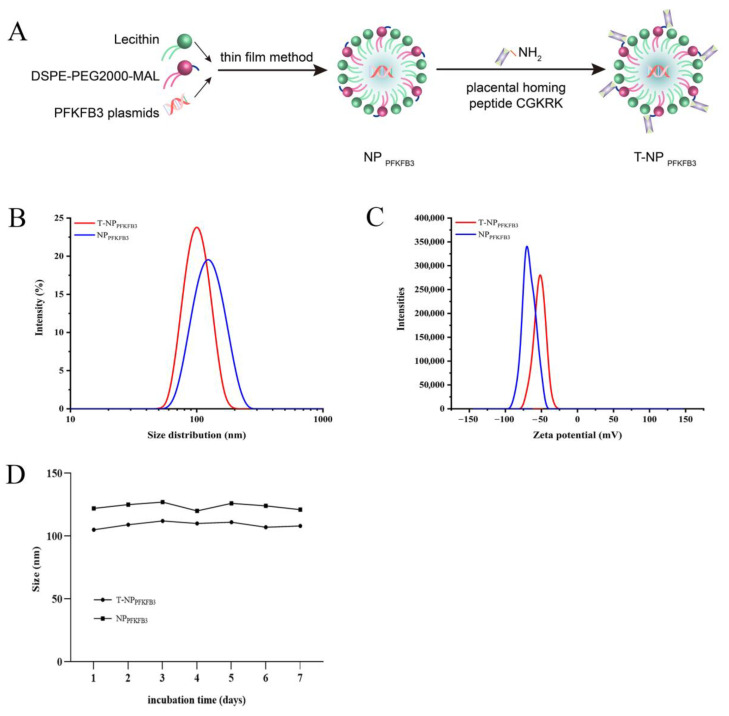
Synthesis and characterization of placenta-targeted NPs loaded with PFKFB3 plasmids: (**A**) Schematic diagram of the synthesis of NPs via the thin film method and the synthesis of T-NP_PFKFB3_ by conjugating the placental homing peptide CGKRK to the surface of NP_PFKFB3_; (**B**) size distributions of NPs as measured by DLS; (**C**) zeta potentials of NPs as determined by DLS; (**D**) effect of incubation in a 10% FBS solution on NP size.

NP_PFKFB3_ was constructed with DSPE-PEG2000-MAL, lecithin, and PFKFB3 overexpression plasmids via the thin film method, and T-NP_PFKFB3_ was prepared by conjugating the placental homing peptide CGKRK to the surface of NP_PFKFB3_ (Figure 1A). We next determined the physical properties of these NPs. Their size distributions and zeta potentials were examined by DLS, revealing average sizes of approximately 122 nm and 105 nm for NP_PFKFB3_ and T-NP_PFKFB3_, respectively (Figure 1B). The average zeta potentials, which represent the nature of the electrostatic potential near the surface of a particle (a value less than −15 mV usually represents the onset of agglomeration, and higher absolute values are correlated with more stable particles), were −63.3 mV and −48.7 mV for NP_PFKFB3_ and T-NP_PFKFB3_, respectively (Figure 1C). These NPs were thus predicted to be highly stable. Next, we dispersed the NPs in a 10% FBS solution and evaluated their stability in serum by detecting the change in particle size. The particle sizes of NP_PFKFB3_ and T-NP_PFKFB3_ remained close to their initial sizes within one week (Figure 1D). In conclusion, these NPs had good stability and were suitable for in vivo experiments.

### 3.2. T-NP_PFKFB3_ Selectively Accumulates in the Mouse Placenta and Upregulates PFKFB3 Expression

**Figure 2:**
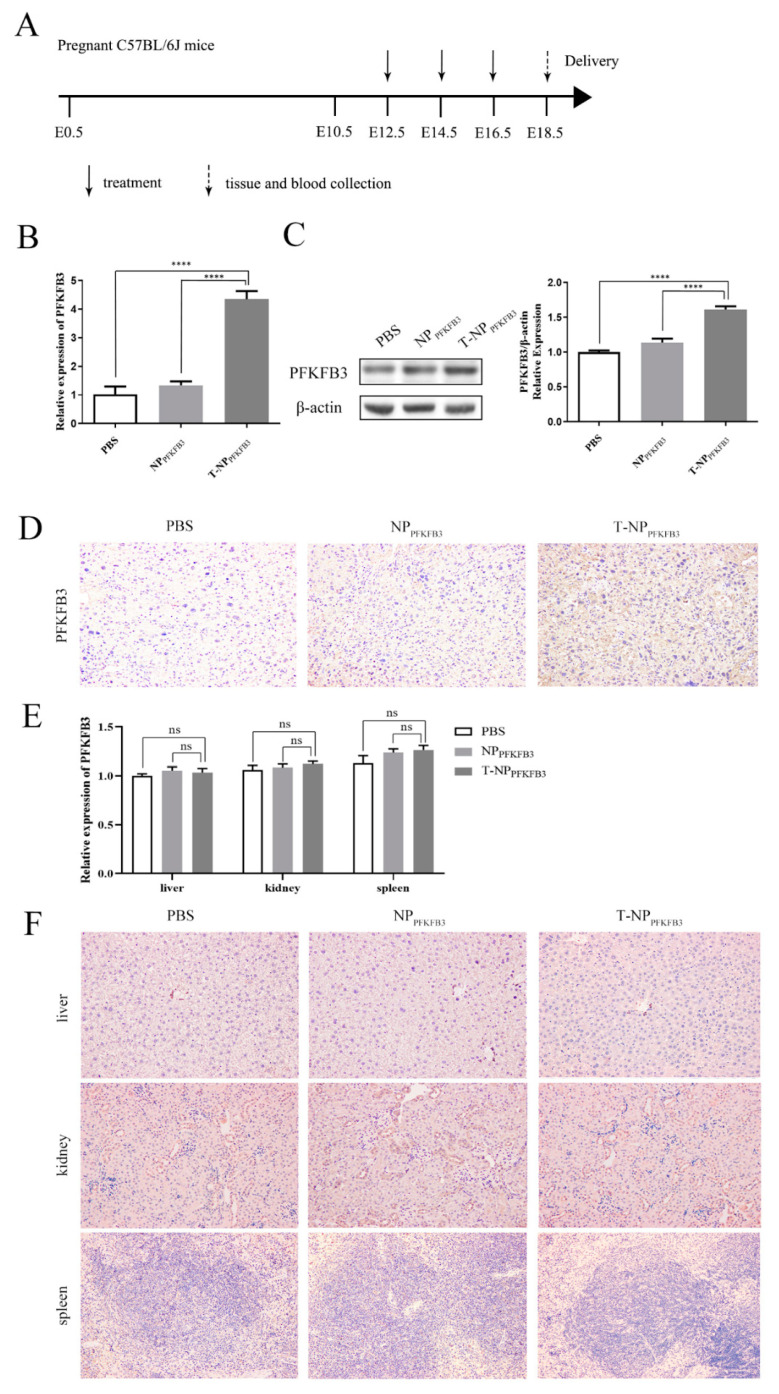
T-NP_PFKFB3_ selectively accumulates in the mouse placenta and upregulates PFKFB3 expression. Pregnant C57BL/6J mice were intravenously injected with PBS, NP_PFKFB3_, or T-NP_PFKFB3_. (**A**) Experimental design to assess the expression of PFKFB3 in pregnant C57BL/6J mice. (**B**) PFKFB3 mRNA expression in placental tissues. (**C**) Representative WB images of PFKFB3 in placentas. (**D**) Representative IHC images of PFKFB3 in placentas. (**E**) PFKFB3 mRNA expression in important maternal organs (liver/kidney/spleen). (**F**) Representative IHC images of PFKFB3 expression in important maternal organs (liver/kidney/spleen). All data were obtained from three mice per group. **** *p* < 0.0001 using one-way ANOVA. n.s.—not significant.

We used a mouse model to assess the functions of the NPs. Pregnant C57BL/6J mice were divided into three groups and intravenously injected with PBS, NP_PFKFB3_, or T-NP_PFKFB3_ (*n* = 8 per group). The experimental design is shown in Figure 2A. We first detected the relative mRNA levels of PFKFB3 in the placentas of mice in the three groups by RT-qPCR to determine whether PFKFB3 accumulated successfully in the placentas. The expression of PFKFB3 in the T-NP_PFKFB3_ group was significantly higher than that in the NP_PFKFB3_ and PBS groups (Figure 2B). We also detected the protein expression of PFKFB3 in these mice by Western blot (WB) and IHC analyses; the representative images are shown in Figure 2C,D. Similarly, the protein expression of PFKFB3 was significantly increased in the placentas of mice in the T-NP_PFKFB3_ group. Then, we collected important maternal organs (liver/kidney/spleen) from three mice in each group. Each mouse includes one liver, two kidneys, and one spleen. Next, RT-qPCR and IHC were performed to assess the accumulation of PFKFB3 in these vital organs, revealing that PFKFB3 expression was not significantly different among the three groups (Figure 2E,F). In conclusion, T-NP_PFKFB3_ can specifically aggregate in the mouse placenta and significantly upregulate the expression of PFKFB3.

### 3.3. T-NP_PFKFB3_ Promotes Placental Angiogenesis and Increases the Placental and Fetal Weights in Mice

**Figure 3:**
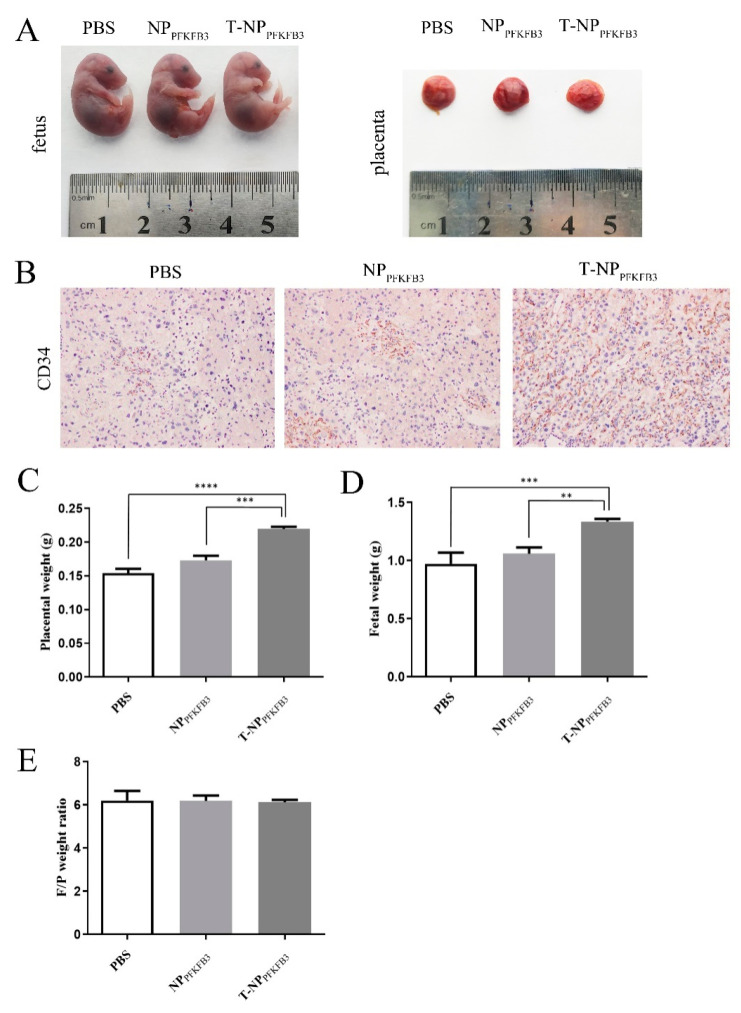
T-NP_PFKFB3_ promotes placental angiogenesis and increases the placental and fetal weights in mice. Pregnant mice were treated with PBS, NP_PFKFB3_, or T-NP_PFKFB3_. Thereafter, they were sacrificed at E18.5 and photographed, and the following data were collected: (**A**) representative images of a mouse placenta and fetus; (**B**) representative image of the placental microvessel density; (**C**) placental weight; (**D**) fetal weight; (**E**) fetal/placental weight ratio. All data were obtained from three litters of mice per group. ** *p* < 0.01, *** *p* < 0.001, and **** *p* < 0.0001 using one-way ANOVA.

PFKFB3 has been shown to be widely expressed in different tissues and organs and to play an important role in regulating angiogenesis [23,24]. Therefore, we hypothesized that the targeted delivery of PFKFB3 to the placenta would promote angiogenesis and thus enhance placental function, and that an increased placental weight would lead to a corresponding increase in fetal weight. We injected PBS, NP_PFKFB3_, and T-NP_PFKFB3_ intravenously into healthy pregnant mice and then collected their placentas and fetuses on E18.5 to compare the differences among the three groups (Figure 3A).

First, to study the effect of PFKFB3 on placental vessels, we performed IHC to detect the expression of CD34 (a vascular EC marker), where positively stained dark brown ring-like structures were indicative of placental microvessels (Figure 3B). The microvessel density (MVD) of the T-NP_PFKFB3_ group was significantly higher than that of the NP_PFKFB3_ and PBS groups, suggesting that the overexpression of PFKFB3 in the placenta promotes angiogenesis. We then measured the placental and fetal weights to assess the effect on placental function. These data were obtained from three litters of mice in each group. The number of mice born per pregnancy ranged from 5 to 9, with 9, 8, and 7 in the PBS group; 9, 5, and 8 in the NP_PFKFB3_ group; and 8, 9, and 6 in the T-NP_PFKFB3_ group. Similarly, compared with those in the NP_PFKFB3_ and PBS groups, the average placental and fetal weights in the T-NP_PFKFB3_ group were effectively increased (Figure 3C,D). In addition, fewer of the smallest (lowest weight) pups were observed in the T-NP_PFKFB3_ group compared to the other groups, thereby significantly reducing the variance in the fetal/placental weight ratio (Figure 3E). Therefore, PFKFB3 promotes placental angiogenesis and placental and fetal growth in mice.

### 3.4. T-NP_PFKFB3_ Has No Obvious Side Effects

**Figure 4:**
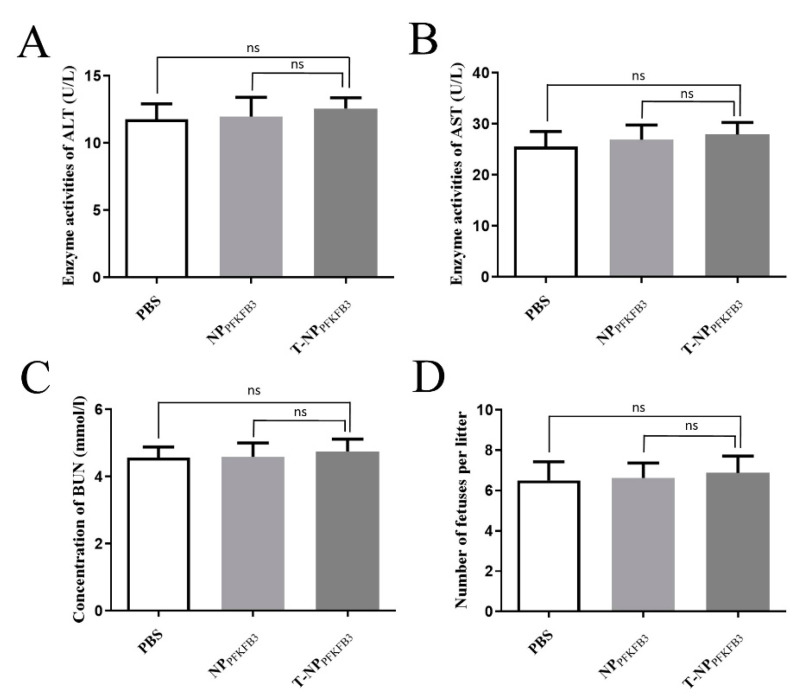
T-NP_PFKFB3_ has no obvious side effects. Enzymatic activities of ALT (**A**) and AST (**B**), the concentrations of BUN (**C**), and the numbers of newborn pups (**D**) on E18.5 of gestation in mice injected with PBS, NP_PFKFB3_, or T-NP_PFKFB3_. All data were obtained from three mice per group. n.s.—not significant.

We further studied the toxic effects of T-NP_PFKFB3_ on mice. Changes in ALT and AST are widely used to evaluate the degree of liver damage [25,26], while the level of BUN indicates the degree of renal damage [27,28]. Changes in the serum levels of ALT, AST, and BUN in mice injected with PBS, NP_PFKFB3_, and T-NP_PFKFB3_ were detected by the appropriate assay kits; no significant difference in their levels were detected among the T-NP_PFKFB3_, NP_PFKFB3_, and PBS groups (Figure 4A–C). This result indicated that the intravenous injection of T-NP_PFKFB3_ did not damage the liver or kidney functions of the mice. In addition, T-NP_PFKFB3_ treatment caused no significant fetal abnormalities and did not alter the average litter size (Figure 4D), indicating a very low toxicity of T-NP_PFKFB3_ in mothers and fetuses. Therefore, T-NP_PFKFB3_ has no obvious side effects.

## 4. Discussion

Pregnancy complications are common, and effective treatment strategies are lacking. At present, no effective therapeutic agents have been approved for the treatment of placental insufficiency or for reducing maternal and neonatal complications, mainly due to the safety and economic benefits of the treatments [20]. Therefore, patients with common but life-threatening pregnancy complications such as preeclampsia (PE) and FGR have no treatment options other than emergency cesarean section; new therapies that can safely enhance placental function and improve the prognosis of mothers and infants are urgently needed [29]. In this study, we first constructed the nanomedicine agent T-NP_PFKFB3_ and assessed its characteristics. Further in vivo experiments showed that intravenously injected T-NP_PFKFB3_ could selectively accumulate in the mouse placenta and upregulate the expression of PFKFB3 without affecting its expression in other important organs (liver/kidney/spleen). T-NP_PFKFB3_ treatment increased the placental MVD and fetal and placental weights in pregnant mice. Finally, we assessed the safety of T-NP_PFKFB3_ and found that compared with those in the NP_PFKFB3_ and PBS groups, the serum levels of ALS/AST/BUN in pregnant mice from the T-NP_PFKFB3_ group were not significantly different; moreover, T-NP_PFKFB3_ caused no obvious fetal abnormalities and did not alter the average litter number. Based on these findings, we believe that T-NP_PFKFB3_ can promote angiogenesis and enhance placental function without obvious side effects. This nanomedicine may serve as a new treatment for pregnancy complications in the future.

In recent years, PFKFB3, a key regulator of glycolysis, has been shown to play an increasingly important role in regulating angiogenesis through metabolic pathways independent of genetic signals [14,24]. De Bock et al. [10] showed that PFKFB3-mediated glycolysis could directly regulate vascular sprouting and promote angiogenesis, and that inactivation of the PFKFB3 gene could inhibit angiogenesis in mice [11]. We also previously demonstrated the role of the MALAT1/miR-26/PFKFB3 axis in the pathogenesis of early-onset preeclampsia (EOPE) and proposed that targeting PFKFB3 to drive glycolysis is a promising therapeutic strategy for EOPE [15]. Therefore, we believe that the overexpression of PFKFB3 in the placenta can promote angiogenesis and enhance placental function, and thus can be used for the treatment of pregnancy complications. However, some limitations have limited the use of PFKFB3 as a therapeutic, such as the fact that PFKFB3 plasmids cannot be targeted to specific sites. Therefore, we herein combined PFKFB3 with a nanomaterial to target the placenta.

Research on nanodrugs for the treatment of pregnancy complications has increased [30]. NPs can function as delivery systems and have many advantages; for example, they can increase drug stability, improve bioavailability, and promote targeted and localized drug release, and they have low toxicity and few side effects [31,32]. NPs are widely used in the treatment of cancer and degenerative diseases [33,34], and common nanomaterials (such as gold, silver, quantum dots, and iron oxide NPs) easily cross the placental barrier and can be detected in fetal circulation, thereby potentially affecting normal placental function [19,35,36]. Liposomes are biodegradable and nontoxic phospholipid-based NPs that have been proven to be capable of delivering insulin-like growth factor-2 (IGF-2) and microRNA inhibitors to the placenta and to improve pregnancy outcomes [21,22]. In addition, after intravenous injection in mice, although the placenta can freely contact the drug in the maternal blood circulation, only a small number of nontargeted NPs are transported to the placenta, and the NPs are often more concentrated in the liver [20,37]. Therefore, it is important to design targeted NPs for maternal and infant medicine. The carrier surface can be modified with specific targeting ligands to increase the specific binding and uptake of particles in cells or tissues [38]. King et al. [21] confirmed that the placenta homing peptide CGKRK could effectively target the placenta and bind to vascular ECs in mice. Therefore, we constructed placenta-targeting CGKRK-modified liposomes as a biocompatible nanocarrier to selectively transport the PFKFB3 plasmid to the placenta and promote angiogenesis. In our study, the results show that T-NP effectively acts on the placenta, while levels of PFKFB3 expression in other important organs (spleen, liver, and kidney) show no significant differences. When constructing the PFKFB3 overexpression plasmid, we chose the pEX-2 vector containing the CMV promoter. The CMV promoter is the most widely used promoter at present; however, some studies show that the expression efficiency of the CMV promoter varies among tissues, and the specific mechanism is still unclear [39,40]. Different studies have reached different conclusions. For example, Furth et al. [39] showed that CMV promoters are highly expressed in the heart, stomach, and spleen but less expressed in the liver, while Arita et al. [41] showed that CMV promoters have higher transcriptional activity in liver cells than RSV and EF1α. In this study, we could not rule out that the reason why there is no significant difference in the expression of the CMV promoter in the examined tissues (spleen, liver, and kidney) is that the CMV promoter is weak in these tissues. Therefore, in a follow-up study, we will change the experimental grouping (such as increasing the EF1α and CAG promoter groups), compare their expression in different tissues to further determine the targeting of the nanodrug, and optimize the construction scheme of the vector. At the same time, to be transferred to clinical practice, we still need further research to optimize NPs, improve their encapsulation efficiency and drug loading, and test the optimal dose targeting the placenta.

Because of their potential use in pregnant women, the safety of NPs is very important. Although many studies have demonstrated the toxicologies of NPs in different species, only a few studies have examined the effects of maternal exposure to NPs on fetal growth and development [42]. In our study, we used mice as an in vivo model to evaluate the safety of NP by evaluating their liver and kidney functions and determining the average litter size. Compared with the PBS group, the mice intravenously injected with T-NP_PFKFB3_ exhibited no obvious abnormalities, indicating that the T-NP_PFKFB3_ nanodrug is a safe strategy for delivering PFKFB3 plasmids to the mouse placenta. Mice are widely used as animal models to study the safety of NPs because of their blood-villiform placenta, which is similar in structure to the human placenta and is easy to surgically examine [43,44]. However, the placental organ has obvious species differences and should thus be studied in a variety of pregnancy animal models in the future. For example, the placental development and pregnancies of sheep and nonhuman primates are closer to those of humans than mice and should thus be used to more accurately simulate the human reproductive system and ensure the safety of NPs.

In conclusion, T-NP_PFKFB3_ injected intravenously can be used to deliver the PFKFB3 overexpression plasmid specifically to the mouse placenta and promote angiogenesis, thereby enhancing placental function and increasing the placenta and fetal weights. Moreover, mothers and fetuses treated with T-NP_PFKFB3_ exhibited no obvious abnormalities; thus, this nanomedicine has potential as a new strategy for the treatment of pregnancy-related complications.

## Data Availability

Not applicable.
